# Interferon-λ Improves the Efficacy of Intranasally or Rectally Administered Influenza Subunit Vaccines by a Thymic Stromal Lymphopoietin-Dependent Mechanism

**DOI:** 10.3389/fimmu.2021.749325

**Published:** 2021-09-29

**Authors:** Liang Ye, Daniel Schnepf, Annette Ohnemus, Li Ching Ong, Hans Henrik Gad, Rune Hartmann, Nils Lycke, Peter Staeheli

**Affiliations:** ^1^ Department of Immunology, International Cancer Center, Shenzhen University Health Science Center, Shenzhen, China; ^2^ Institute of Virology, Medical Center University of Freiburg, Freiburg, Germany; ^3^ Department of Microbiology and Immunology, University of Gothenburg, Gothenburg, Sweden; ^4^ Department of Molecular Biology and Genetics, Aarhus University, Aarhus, Denmark

**Keywords:** interferon-λ, TSLP pathway, influenza a virus, mucosa, intestinal immunity

## Abstract

Previous work showed that interferon-λ (IFN-λ) can trigger the synthesis of thymic stromal lymphopoietin (TSLP) by specialized epithelial cells in the upper airways of mice, thereby improving the performance of intranasally administered influenza vaccines. Here we demonstrate that protein-only influenza vaccines containing either IFN-λ or TSLP boosted antigen-specific IgG1 and IgA responses and enhanced the resistance of mice to influenza virus challenge, irrespective of whether the vaccines were applied *via* the intranasal or the rectal route. TSLP receptor deficiency negatively influenced vaccine-induced antiviral immunity by impairing the migration of dendritic cells from the airways to the draining lymph nodes of immunized mice, thereby restraining follicular helper T cell and germinal center B cell responses. As previously observed during intranasal vaccination, the adjuvant effect of IFN-λ on a rectally administered influenza vaccine was no longer observed when TSLP receptor-deficient mice were used for immunization, highlighting the central role of the IFN-λ/TSLP axis for vaccine-induced antiviral immunity in the mucosa.

## Introduction

Influenza viruses cause severe respiratory disease in humans, resulting in approximately 300,000 deaths each year worldwide (1, 2). Vaccination with subunit vaccines can ameliorate influenza virus-induced disease, but vaccine responses are usually weak in the most vulnerable population, including the elderly (3, 4). This problem might be solved in the near future by supplementing viral subunit vaccines with adjuvants that promote robust immune responses (5). Delivering viral subunit vaccines by intranasal or rectal routes that specifically trigger mucosal immune responses might further enhance the potency of influenza vaccines (1, 6).

Thymic stromal lymphopoietin (TSLP) is a cytokine of the interleukin-7 (IL-7) family that is predominantly produced by epithelial cells in mucosal tissues (7). TSLP signals through a heterodimeric receptor complex composed of the TSLP receptor (TSLPR) and the IL-7 receptor α-chain (IL-7Rα) in humans and mice (7, 8). TSLP acts by binding to TSLPR expressed on immune cells, including dendritic cells (DCs), T cells and B cells (8). TSLP can stimulate the proliferation of B and T cell progenitors (9, 10). Recent studies indicate that viral infection can induce TSLP expression in upper airway microfold (M) cells, and that expression of TSLP correlates with viral load after influenza virus infection (11–13). These results suggested that TSLP might play a role in regulating adaptive antiviral immune responses. Indeed, TSLP can enhance antiviral CD8^+^ T cell responses by acting on inflammatory and migratory DCs (12). TSLP can also induce human T follicular helper (Tfh) cell differentiation and IgE production by activating DCs to express OX40 ligand (14).

Dysregulated production of TSLP can result in disease. For example, overproduction of TSLP in the skin is associated with atopic dermatitis, contact allergy and lupus skin lesions in humans (15). TSLP-specific antibodies are used to ameliorate disease in asthma patients (7, 16, 17). TSLP can initiate allergic airway inflammation in mice (18). Overproduction of TSLP can also be protective, suppressing breast cancer development and preventing pancreatic cancer in mice by a mechanism that involves CD4 T cells (19).

TSLP can exert adjuvant activity on protein vaccines (13, 20, 21). TSLP was shown to boost production of antigen-specific systemic IgG and mucosal IgA in mice immunized by the intranasal route with HIV glycoprotein gp140 (20). Mice lacking functional TSLPR showed diminished antigen-specific IgA responses after intranasal immunization with a pneumococcal surface protein (21). We demonstrated that TSLP can boost antibody production and T cell responses, and we showed that TSLP is the crucial factor that mediates the strong adjuvant activity of IFN-λ on mucosal influenza vaccines (13). IFN-λ stimulated the migration of CD103^+^ DCs to draining lymph nodes in the mouse model, stimulating the germinal center reaction and enhancing vaccine-specific systemic IgG1 and mucosal IgA levels, which resulted in efficient protection from influenza virus challenge and strongly reduced transmission of virus to naive contact animals (13). No such vaccine adjuvant activity of IFN-λ was observed in *Tslpr^-/-^* mice (13), demonstrating that endogenous TSLP signaling determines the immunity-enhancing effect of IFN-λ.

IFN-λ can regulate antiviral immunity mediated by CD8^+^ T cells (13, 22, 23). CD8^+^ T cell responses were blunted in mice lacking a functional IFN-λ system, facilitating reinfection with influenza A viruses (22). This protective effect was due to IFN-λ acting directly on migratory dendritic cells (22). Infection with a live-attenuated influenza virus that strongly boosts the production of endogenous IFN triggered robust CD8^+^ T cell responses in wild-type but not *Tslpr^-/-^* mice (13), suggesting that the IFN-λ/TSLP axis is also regulating cytotoxic T cell responses after viral insults of the respiratory tract.

Although the IFN-λ/TSLP axis might be employed to improve the efficacy of existing influenza vaccines, current evidence that the TSLP system is of crucial importance for vaccine-induced protective immunity is quite limited. In this study, we demonstrate that recombinant TSLP exhibited strong adjuvant activity on various intranasal influenza vaccines. Immunization of *Tslpr^-/-^* mice with influenza subunit vaccines did not result in robust protection against challenge with virulent influenza virus. This lack of protection was associated with low numbers of CD103^+^ DCs in draining lymph nodes of immunized mice, low numbers of T follicular helper (Tfh) cells and poor vaccine-induced germinal center B cell responses. Interestingly, IFN-λ or TSLP alone also improved the performance of influenza vaccines applied by the rectal route. TSLP signaling was essential for the enhancing effect of IFN-λ on rectally administered influenza vaccines, illustrating the importance of the IFN-λ/TSLP axis for mucosal protective immunity in general.

## Results

### TSLP Enhances Vaccine-Induced Immunity Against Influenza A Virus-Induced Disease

**Figure 1:**
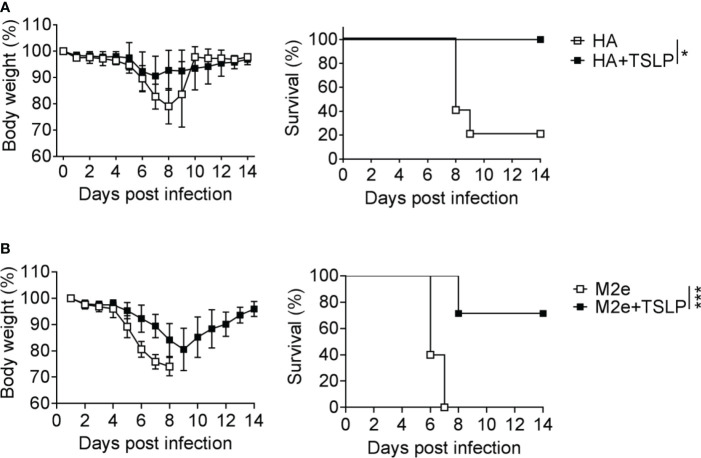
TSLP boosts efficacy of influenza vaccines administered by the intranasal route. **(A)** B6-WT mice (n = 5-6 per group) were immunized by intranasal application of the Influsplit Tetra^®^ vaccine (HA) in the presence or absence of 1 μg TSLP. Three weeks after the third booster immunization, mice were challenged with 3×10^3^ PFU of influenza A virus PR8 strain. Weight loss and survival were monitored daily for 14 days. **(B)** B6-WT mice (n = 5-7 per group) were intranasally immunized with the CTA1-3M2e-DD (M2e) vaccine in the presence or absence of 1 μg TSLP. Five weeks after a single booster immunization, mice were challenged with 50 PFU of influenza A virus PR8 strain. Weight loss and survival were monitored daily for 14 days. Mice were sacrificed as soon as they lost 25% of their original body weight. Data are shown as mean ± SD. *P < 0.05, ***P < 0.001, by log-rank test. Data are representative of two independent experiments.

We previously showed that TSLP can boost influenza vaccine-induced serum IgG1 titers in mice (13), but it remained unclear whether enhanced antibody titers in TSLP-treated mice would provide better protection from influenza virus-induced disease. To address this issue, we first performed intranasal immunizations of WT mice with Influsplit Tetra^®^ (designated HA vaccine in this study), a commercial influenza vaccine that contains particle-derived hemagglutinin (HA) of four different seasonal influenza viruses as its main component. One group of animals was immunized with non-supplemented HA vaccine, whereas the other group received HA vaccine supplemented with 1 µg TSLP. After three booster immunizations, all animals were infected with a lethal dose of influenza A virus strain PR8/34. Under these conditions, mice immunized with the non-supplemented HA vaccine started to lose weight on day 6 post-infection, and 80% of the animals had to be euthanized between day 8 and day 9 (**Figure 1A**). In contrast, weight loss of mice immunized with the TSLP-supplemented HA vaccine was minimal and all animals survived the challenge infection (**Figure 1A**).

A similar picture emerged when WT mice were immunized with CTA1-3M2e-DD, a fusion protein that contains the highly conserved ectodomain of the influenza A virus M2 protein (designated M2e vaccine in this study) (24). Mice immunized twice with TSLP-supplemented M2e vaccine by the intranasal route lost substantially less weight after infection with PR8/34 than control mice immunized with plain M2e vaccine (**Figure 1B**). The majority of mice immunized with the TSLP-containing M2e vaccine survived the challenge infection whereas all control animals had to be euthanized due to severe symptoms and massive weight loss (**Figure 1B**). Taken together, these results demonstrated that TSLP enhances protective antiviral immunity induced by influenza subunit vaccines applied by the intranasal route.

### Vaccine-Induced Influenza Immunity Is Strongly Reduced in *Tslpr* ^-/-^ Mice

**Figure 2:**
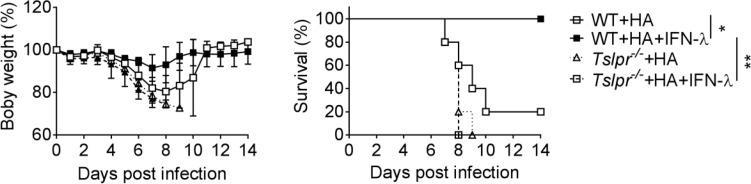
TSLP receptor deficiency negatively affects performance of intranasal influenza vaccines. B6-WT (n = 5) and *Tslpr^–/–^* (n = 5) mice were intranasally immunized with Influsplit Tetra^®^ vaccine (HA) in the presence or absence of 1 μg IFN-λ2. Four weeks after the third booster immunization, mice were infected with 3×10^3^ PFU of influenza A virus strain PR8/34. Weight loss and survival were monitored daily for 14 days. Mice were sacrificed as soon as they lost 25% of their original body weight. *P < 0.05, **P < 0.01, by log-rank test. Data are representative of two independent experiments and shown as mean ± SD.

Our previous work showed that IFN-λ is unable to boost influenza vaccine-induced IgG1 and IgA levels in *Tslpr^-/-^* mice (13), but it remained unclear whether *Tslpr^-/-^* mice would still profit from IFN-λ-adjuvanted vaccines. To answer this question, we immunized WT and *Tslpr^-/-^* mice with the HA vaccine by the intranasal route. Four cycles of immunization were performed using either plain vaccine or vaccine supplemented with 1 µg IFN-λ. After infection with influenza A virus strain PR8/34, we observed a rapid weight loss in WT and *Tslpr^-/-^* mice immunized with the non-adjuvanted HA vaccine, and most of these mice had to be killed for animal welfare reasons before day 10 post-infection (**Figure 2**). As expected, weight loss in WT mice immunized with the HA vaccine supplemented with IFN-λ was minimal, and all animals survived the challenge infection (**Figure 2**). In contrast, all *Tslpr^-/-^* mice that were immunized with the IFN-λ-supplemented HA vaccine showed rapid weight loss after influenza virus challenge, and these mice had to be euthanized within 8-9 days post-infection (**Figure 2**). Control experiments with naïve mice demonstrated that WT and *Tslpr^-/-^* mice exhibit similar intrinsic resistance to infection with PR8 (**Supplementary Figure 1**). Since previous work had shown that WT and *Tslpr^-/-^* mice display similar antibody responses after intranasal immunization with non-adjuvanted or type I IFN-adjuvanted vaccines (25), these findings collectively demonstrate that functional TSLP receptors are specifically required for optimal efficacy of IFN-λ-adjuvanted mucosal vaccines.

### TSLPR Deficiency Impairs Germinal Center Reaction in Lymph Nodes of Immunized Mice

**Figure 3:**
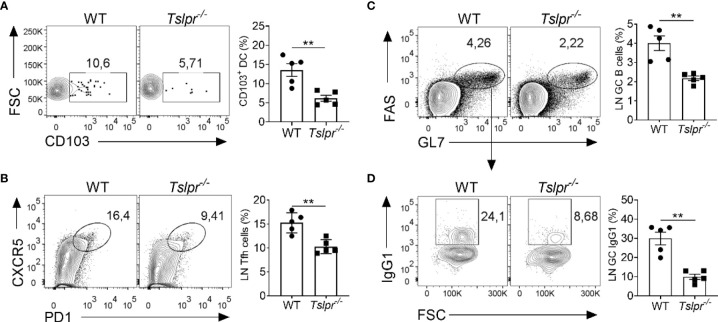
TSLPR deficiency impairs migration of DCs and limits germinal center reaction in draining lymph nodes of mice infected with live-attenuated influenza virus. **(A)** B6-WT (n = 5) and *Tslpr^–/–^* mice (n = 5) were intranasally infected with live-attenuated influenza A virus strain hvPR8-ΔNS1, and the frequency of CD103^+^ DCs among live CD11c^+^MHC-II^+^ immune cells were determined at day 5 post-infection. **(B–D)** B6-WT (n = 5) and *Tslpr^–/–^* mice (n = 5) were intranasally infected with hvPR8-ΔNS1. Mice were sacrificed on day 10 post-infection and cells in draining lymph nodes were analyzed by flow cytometry for **(B)** Tfh cells among live CD19^−^CD4^+^ CD44^+^ cells. **(C)** Germinal center B cells among live CD4^-^B220^+^ cells or **(D)** IgG1^+^ germinal center B cells among live CD4^−^B220^+^GL7^+^Fas^+^ cells. Each symbol represents the result from an individual animal. **P < 0.01 by unpaired two-tailed Student’s t-test. Results are representative of three independent experiments and shown as mean ± SEM.

To better understand the mechanism by which TSLP improves the efficacy of influenza vaccines, we monitored early immunological events by flow cytometry in draining lymph nodes of WT and *Tslpr^-/-^* mice infected with hvPR8-ΔNS1, a live-attenuated influenza A virus that cannot suppress cytokine production of the infected host (13, 26, 27). We found that draining lymph nodes of *Tslpr^-/-^* mice contained about 2-fold fewer CD103^+^ migratory DCs than WT mice on day 5 post-infection (**Figure 3A**). The frequency of CXCR5^+^ PD1^+^ Tfh cells among the CD19^−^CD4^+^ CD44^+^ cells was also significantly reduced in lymph nodes of *Tslpr^-/-^* mice on day 10 post-infection compared with WT mice (**Figure 3B**). Furthermore, the frequency of FAS^+^ GL7^+^ germinal center B cells among live CD4^-^B220^+^ cells was about 2-fold lower in lymph nodes of *Tslpr^-/-^* mice compared with WT mice (**Figure 3C**), and the frequency of IgG1^+^ germinal center B cells among live CD4^−^B220^+^GL7^+^Fas^+^ cells in the lymph nodes was approximately 3-fold reduced (**Figure 3D**). Similar differences between *Tslpr^-/-^* and WT mice were observed when the frequencies of splenic CD103^+^ migratory DCs, Tfh cells and germinal center B cells of hvPR8-ΔNS1-infected mice were compared (**Supplementary Figure 2**). Importantly, these populations were not reduced at baseline in lymph nodes and spleens of *Tslpr^-/-^* mice (**Supplementary Figure 3**). Taken together, these results demonstrated that TSLP promotes the germinal center reaction in lymphoid organs of immunized mice.

### TSLP and IFN-λ Can Boost the Efficacy of Rectally Administered Influenza Vaccines

**Figure 4:**
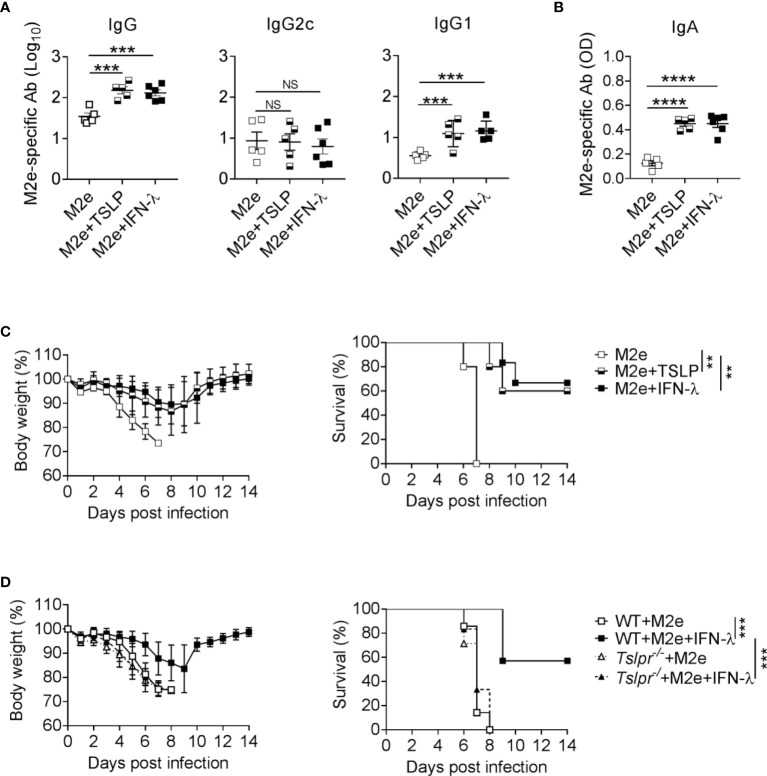
The IFN-λ/TSLP axis enhances the efficacy of rectally administered M2e vaccine. **(A–C)** B6-WT mice were immunized by the rectal route with CTA1-3M2e-DD (M2e) alone (n = 5), M2e+TSLP (n = 5) or M2e+IFN-λ (n = 6). Ten days later, all mice received a booster immunization. M2e-specific antibodies in **(A)** sera and **(B)** BAL fluid on day 10 after booster immunization were measured by ELISA. Each symbol represents an individual animal. Results are representative of three independent experiments and shown as mean ± SEM. ***P < 0.001 and ****P < 0.0001 by one-way ANOVA with Tukey’s multiple-comparison test. NS, no significant difference. **(C)** Groups of immunized mice were challenged with 50 PFU of influenza A virus strain PR8/34. Weight loss and survival were monitored daily for 14 days. Animals were sacrificed when they lost 25% of their original body weight. Data are shown as mean ± SD. **P < 0.01, by log-rank test. **(D)** B6-WT (n = 7) and *Tslpr^–/–^* (n = 7) mice were rectally immunized with the CTA1-3M2e-DD (M2e) vaccine in the presence or absence of 1 μg IFN-λ. Ten days later, all mice received a booster immunization and, 24 days later, they were infected with 50 PFU of influenza A virus strain PR8/34 as in **(C)**. ***P < 0.001, by log-rank test. Data are pooled from two independent experiments and shown as mean ± SD.

Since IFN-λ exhibits strong adjuvant activity on influenza subunit vaccines when immunizations are performed *via* the intranasal but not the intramuscular route (13), we wondered whether IFN-λ might also have adjuvant activity during rectal immunizations. Rectal vaccination is an effective mucosal vaccine delivery route that can provide powerful protective immunity (28, 29). By this application route, the vaccine should quickly reach epithelia-rich mucosal surfaces like in the case of intranasal vaccine administration. To evaluate this possibility, we applied either 1 µg of plain M2e vaccine or 1 µg of M2e vaccine supplemented with 1 µg IFN-λ to the rectum of WT mice and performed a booster immunization 10 days later. M2e vaccine supplemented with IFN-λ induced approximately 5-fold higher total M2e-specific serum IgG levels and mainly boosted the synthesis of M2e-specific IgG1 antibodies (**Figure 4A**). Including IFN-λ into the rectal vaccine also increased the levels of M2e-specific IgA in BAL fluids (**Figure 4B**). A comparable booster effect was observed when TSLP rather than IFN-λ was used as vaccine adjuvant (**Figure 4A, B**), demonstrating that both cytokines can exert adjuvant effects at multiple mucosal sites and that rectal immunization results in enhanced antigen-specific IgA levels in the respiratory tract.

To determine whether the elevated M2e-specific antibody levels might confer enhanced protection against influenza A virus infection, we challenged the mice with wild-type PR8/34 virus. Mice immunized rectally with plain M2e vaccine rapidly lost weight and had to be euthanized between days 6 and 7 post-infection (**Figure 4C**). In contrast, mice rectally immunized with M2e in the presence of IFN-λ or TSLP showed less severe weight loss and most of these animals survived the challenge infection (**Figure 4C**).

To investigate whether the adjuvant activity of IFN-λ on rectally administered M2e vaccine was dependent on TSLP as previously described for intranasal applications (13), we determined whether *Tslpr^-/-^* mice would benefit from rectal immunization with IFN-λ-adjuvanted M2e vaccine. We observed that all immunized *Tslpr^-/-^* mice succumbed to infection with influenza A virus strain PR8/34, whereas the majority of WT mice immunized with the IFN-λ-adjuvanted M2e vaccine survived the infection (**Figure 4D**). Taken together, these results demonstrated that IFN-λ and TSLP can improve the performance of rectally administered vaccines, and that the IFN-λ/TSLP axis is operative in the airways and the gastrointestinal tract.

## Discussion

Previous work revealed that IFN-λ can boost the efficacy of intranasal influenza subunit vaccines *via* an indirect mechanism that involves IFN-λ-mediated production of TSLP by specialized epithelial cells in the airways (13). However, it remained unclear whether the IFN-λ/TSLP axis is also operative at other mucosal sites. Here we found that recombinant TSLP can greatly improve the efficacy of intranasal influenza vaccines. Similar immune-enhancing effects of IFN-λ and TSLP were observed if an influenza subunit vaccine was delivered to mice *via* the rectal route. Importantly, as in the airways, TSLP signaling was also required for the vaccine adjuvant activity of IFN-λ in the intestinal tract, demonstrating that the IFN-λ/TSLP axis is operative in both the airways and the gastrointestinal tract. These findings support the view that IFN-λ plays a previously overlooked role as regulator of adaptive immunity at mucosal sites (23, 30).

Immune cells show poor responsiveness to IFN-λ (23, 31). Thus, to alert the immune system about an ongoing viral attack in the mucosa, IFN-λ acts indirectly by using TSLP that can activate a large number of immune cells (30). TSLP is a potent initiator of type 2 inflammatory immune responses at barrier surfaces that, if not regulated stringently, can result in asthma exacerbation (18). TSLP has protective activity under certain conditions and can prevent the development of cancer or enhance resistance to viral infections (11, 19). Previous work further showed that TSLP has vaccine adjuvant activity (13, 20, 21). We confirmed these observations and found that TSLP can activate Tfh cells and can stimulate the germinal center B cell response after infection with a live-attenuated virus by promoting the migration of dendritic cells from the mucosal tissue to the draining lymph nodes. Thus, IFN-λ produced by virus-infected cells exhibits at least two different activities. First, it triggers innate immune responses that help limiting virus spread in the tissue (32). Second, IFN-λ improves adaptive immune responses to invading pathogens by promoting the production of TSLP in epithelia-rich tissues (ref (23). and current work).

Interestingly, *Tslpr^-/-^* and wild-type mice responded similarly well to intranasal immunization with non-adjuvanted or type I IFN-adjuvanted vaccines (25). Furthermore, naive *Tslpr^-/-^* and wild-type mice exhibited a similar degree of susceptibility to infection with non-pathogenic (11) and pathogenic (**Supplementary Figure 1**) influenza A virus strains. Thus, *Tslpr^-/-^* mice appear to specifically lack the ability to profit from the vaccine-improving effects of IFN-λ. Earlier work indicated that *Tslpr^-/-^* mice do not show normal IgA responses after immunization with a cholera toxin-containing vaccine (21). Since this vaccine likely did not trigger IFN-λ production, this observation suggests that additional pathogen-induced cytokines may engage the TSLP system to improve adaptive immunity.

Using influenza virus as a model for severe airway infections, previous work demonstrated that IFN-λ can also improve antiviral immunity mediated by CD8^+^ T cells (13, 22). In one of these studies, a live-attenuated influenza virus mutant was used for immunizations that cannot block cytokine production in the infected host. Under these conditions, *Tslpr^-/-^* mice mounted a much less pronounced antiviral CD8^+^ T cell response compared with wild-type mice (13), indicating that the T cell-stimulatory effect of IFN-λ was mediated by TSLP. Our current work showed that this particular live-attenuated influenza virus failed to trigger efficient migration of CD103^+^ DCs to draining lymph nodes and did not efficiently induce germinal center reactions in *Tslpr^-/-^* mice (**Figure 3**). Consistent with this observation, depletion of DCs dampened the enhancing effects of TSLP on Tfh and GC B cells responses in an unrelated study (33), supporting the notion that TSLP-activated DCs are required for Tfh and GC B responses. We thus speculate that the increased presence of activated CD103^+^ DCs in lymph nodes of mice immunized with IFN-λ-adjuvanted vaccines triggered stronger antigen-specific germinal center reactions and stronger antiviral CD8^+^ T cell responses compared with mice receiving non-adjuvanted vaccines. Two other studies indicated that IFN-λ can enhance protective CD8^+^ T cell responses after DNA vaccination by modulating regulatory T cell activity (34, 35). Since the DNA vaccines were applied intramuscularly, it remains unclear how IFN-λ could be beneficial in these cases and whether the IFN-λ/TSLP axis is involved.

Our previous work revealed that IFN-λ can stimulate TSLP secretion in specialized upper airways epithelial cells, namely M cells, to regulate adaptive mucosal immunity (13). Although M cells are also abundantly present in gut-associated lymphoid tissues, they display a distinct phenotype and exhibit functional specialization distinct from M cells in the upper airway (36). It remains an open question if intestinal M cells or other specialized epithelial cells in the gut respond to IFN-λ by inducing the production of TSLP. A recent study in which single-cell RNA sequencing data of the small intestine were analyzed concluded that tuft cells can express TSLP (37). However, another study found that tuft cells mainly produce IL-25 and are not the major source of TSLP in the small intestine (38). Future single-cell RNA sequencing work may answer the question which epithelial cell types are producing TSLP in response to IFN-λ in the intestinal tract.

Taken together our work revealed that the protective efficacy of protein-only vaccines administered by the mucosal route can be improved dramatically if IFN-λ or TSLP are co-administered as adjuvants. Our work further demonstrated that the vaccine adjuvant effect of IFN-λ in both the airways and the intestinal tract are strongly dependent on a functional TSLP system.

## Materials and Methods

### Mice

8-10 week-old female C57BL/6 mice (designated B6-WT) were purchased from Janvier Laboratories (Strasbourg, France). *Tslpr^–/–^* mice with C57BL/6 genetic background were described before (13). All animals used in this study were maintained under specific pathogen-free conditions in the animal facility of the Institute of Virology at the University of Freiburg.

### Immunization With M2 Vaccine by Intranasal or Rectal Routes

Mice were anesthetized with a ketamine/xylazine mixture and immunized with 1 μg of the CTA1-3M2e-DD (M2e) vaccine (24) alone, 1 μg of M2e plus 1 μg IFN-λ2 (13, 39), or 1 μg of M2e plus 1 μg TSLP (555-TS, R&D Systems) (39). Intranasal immunizations were performed by applying the vaccines in a 40 µl volume to the nostrils. Immunizations by the rectal routes were performed by applying 50 µl of the M2e vaccine using a syringe with button cannula. Booster immunizations were performed 10 days later. Blood and BAL fluid samples were collected at day 10 post booster immunization and M2e-specific antibodies titers were analyzed by ELISA.

### Intranasal Immunization With Influsplit Tetra^®^ Vaccine

B6-WT and *Tslpr^–/–^* mice were immunized by intranasal application of 4 μg of Influsplit Tetra^®^ (GSK) vaccine (containing 1 μg HA of each seasonal influenza virus subtype, designated here “HA”) alone, or 4 μg of HA plus 1 μg IFN-λ2, or 4 μg of HA plus 1 μg TSLP. Immunizations were performed by applying the vaccines in a 40 µl volume to the nostrils. Booster immunizations were performed 10, 20 and 30 days later. Subsequently, immunized animals were anesthetized with a ketamine/xylazine mixture and infected by applying 3×10^3^ PFU of PR8/34 influenza A virus to the nostrils in a volume of 40 µl. Changes in body weight were monitored for 14 days. Mice were sacrificed when 75% of the initial body weight was reached.

### Immunization by Infection With Live-Attenuated Influenza A Virus

B6-WT and *Tslpr^–/–^* mice were anesthetized with a ketamine/xylazine mixture and infected intranasally with 10^5^ PFU of live-attenuated influenza A virus strain hvPR8-ΔNS1 (26) that cannot block cytokine production by the infected host in a volume of 40 μl.

### Challenge Infections With Influenza A Virus

Influenza A virus strain PR8/34 was applied in a volume of 40 µl to the nostrils of animals anesthetized with a ketamine/xylazine mixture. For mice immunized with the M2e vaccine, the challenge dose was 50 PFU. For mice immunized with the HA vaccine, the challenge dose was 3x10^3^ PFU. Changes in body weight were monitored for 14 days. Mice were sacrificed as soon as they had lost 25% of their initial body weight.

### Measuring M2e-Specific Antibody Levels

M2e-specific antibodies in serum and BAL fluids of immunized mice were determined by ELISA as described (13, 25). Briefly, high-binding 96-well microtiter plates (MaxiSorp, Nunc) were coated with 2 µg/ml of M2e peptide (GenScript) and incubated overnight at 4°C. Next, the ELISA plates were washed four times with wash buffer (PBS containing 0.1% Tween 20) and blocked with 5% BSA in PBS for 2 h at 37°C. Plates were washed four times with wash buffer. Afterwards, diluted serum and BAL fluid samples were added and incubated for 1 h at room temperature. Plates were washed three times with wash buffer. Horseradish peroxidase-labeled antibodies directed against either total IgG (62-6520, Invitrogen), IgG1 (A10551, Invitrogen), IgG2c (1078-05, Southern Biotech), or IgA (626720, Invitrogen) were added to each well and incubated for 1 h at room temperature. Plates were washed four times and incubated with tetramethylbenzidine (TMB) substrate (Life Technologies) for 10-30 min. The reaction was stopped by adding 0.5 M H_2_SO_4,_ then the absorbance was measured at 450 nm. The endpoint M2e-specific IgG subclasses titers were defined as highest dilutions resulting in optical density (OD) values which were four-fold above background. Undiluted BAL fluids samples were used to measure M2e-specific IgA by ELISA. Since IgA titers were low, the OD readings were plotted directly.

### Antibodies for Flow Cytometry

Different combinations of the following fluorescence-labeled antibodies were used for flow cytometry: For analysis of DC subsets in the lymph nodes a combination of CD103 (2E7, BioLegend), CD11c (N418, BioLegend) anti-CD16/32 (93, BioLegend) and MHC-II (M5/114.15.2, BioLegend) was used. For analysis of Tfh cells and GC B cells in spleen and lymph nodes a combination of anti-CD4 (RM4-5, BioLegend), anti-CD19 (1D3, BioLegend), anti-B220 (RA3-6B2, eBioscience), anti-CD16/32 (93, BioLegend), anti-PD1 (29F.1A12, BioLegend), anti-CXCR5 (L138D7, BioLegend), anti-CD44 (IM7, BioLegend), anti-FAS (SA367H8, BioLegend), anti-IgG1 (A85-1, BD Biosciences) and anti-GL7 (GL7, BioLegend) was used.

### Statistical Analysis

Statistical significance was determined by unpaired two-tailed Student’s t-test, one-way ANOVA with Tukey’s multiple-comparison test, or log-rank test, which was indicated in the relative figure legends. Graph generation and statistical analyses were performed using GraphPad Prism 7 software. Data are represented as mean and SEM, or as mean and SD.

## Data Availability Statement

The raw data supporting the conclusions of this article will be made available by the authors, without undue reservation.

## Ethics Statement

All mouse experiments were carried out in accordance with the Federation for Laboratory Animal Science Associations (FELASA) guidelines and the national animal welfare body, and were approved by the Regierungspräsidium Freiburg animal welfare committee (permits G-16/177 and G-17/74).

## Author Contributions

LY, DS and AO performed experiments. LO, HG, RH, and NL provided essential reagents. LY and PS conceived the project. LY and PS wrote the manuscript with input from all authors. All authors contributed to the article and approved the submitted version.

## Funding

This work was supported by the Deutsche Forschungsgemeinschaft grant agreement STA 338/15-1 to PS, the Danish Council for Independent Research, Medical Research grant agreement 11-107588 to RH and National Natural Science Foundation of China (32170937), Shenzhen Science and Technology Program (RCBS20200714114958310, 20200803131335002), Guangdong medical science and technology research foundation (A2021336), Guangdong Basic and Applied Basic Research Foundation (2020A1515110410, 2020A1515010917), and SZU Top Ranking Project (86000000210) agreement to LY.

## Conflict of Interest

The authors declare that the research was conducted in the absence of any commercial or financial relationships that could be construed as a potential conflict of interest.

## Publisher’s Note

All claims expressed in this article are solely those of the authors and do not necessarily represent those of their affiliated organizations, or those of the publisher, the editors and the reviewers. Any product that may be evaluated in this article, or claim that may be made by its manufacturer, is not guaranteed or endorsed by the publisher.

## Supplementary Material

Supplementary Figure 1 Influenza virus susceptibility of naïve Tslpr-/- and WT mice is comparable. Naive B6-WT (n = 6) and *Tslpr^–/–^* mice (n = 6) were intranasally infected with **(A)** 40 PFU or **(B)** 100 PFU of influenza virus PR8. Weight loss and survival were monitored for 14 days. Mice were sacrificed when having lost 25% of their original body weight. Data are representative of two independent experiments and shown as mean ± SD.

Supplementary Figure 2 TSLPR deficiency impairs migration of DCs and restrains GC reaction in the spleen of mice infected with live-attenuated influenza virus. **(A)** B6-WT (n = 5) and *Tslpr^–/–^* mice (n = 5) were intranasally infected with live-attenuated influenza virus (hvPR8-ΔNS1), and the frequency of CD103^+^ DCs among live CD11c^+^MHC-II^+^ immune cells in the spleen were determined at day 5 post-infection. **(B, C)** B6-WT (n = 5) and *Tslpr^–/–^* mice (n = 5) were intranasally infected with hvPR8-ΔNS1 and sacrificed on day 10 post-infection. The frequencies of **(B)** Tfh cells among live CD19^−^CD4^+^ CD44^+^ cells and (**C**, upper panels) GC B cells among live CD4^-^B220^+^ cells in spleen were determined by flow cytometry. (**C**, lower panels) Flow cytometry analyzing the frequency of IgG1^+^ GC B cells among live CD4^−^B220^+^GL7^+^Fas^+^ cells in the spleen. Data are representative of three independent experiments and shown as mean ± SEM. Each symbol represents the result from an individual animal. **P < 0.01, ***P < 0.001 by unpaired two-tailed Student’s t-test.

Supplementary Figure 3 TSLPR deficiency does not alter the proportions of migratory DCs and has no effect on the GC reaction in the lymph nodes and spleen. **(A)** The frequency of CD103^+^ DCs among live CD11c^+^MHC-II^+^ immune cells from lymph nodes and spleen was determined in naïve B6-WT (n = 6) and *Tslpr^–/–^* mice (n = 5). **(B, C)** B6-WT (n = 6) and *Tslpr^–/–^* mice (n = 5) were sacrificed, and the frequencies of **(B)** Tfh cells among live CD19^−^CD4^+^ CD44^+^ cells and **(C)** GC B cells among live CD4^-^B220^+^ cells in lymph nodes and spleen were determined by flow cytometry. Data are representative of two independent experiments and shown as mean ± SEM. Each symbol represents the result from an individual animal. Unpaired two-tailed Student’s t-tests were performed to determine significant difference. NS, no significant difference.
