# Supplementary figures and images for: Interferon-λ Improves the Efficacy of Intranasally or Rectally Administered Influenza Subunit Vaccines by a Thymic Stromal Lymphopoietin-Dependent Mechanism
Source: Front Immunol. 2021 Sep 29;12:749325. doi: 10.3389/fimmu.2021.749325 (PMC8511795; doi:10.3389/fimmu.2021.749325)

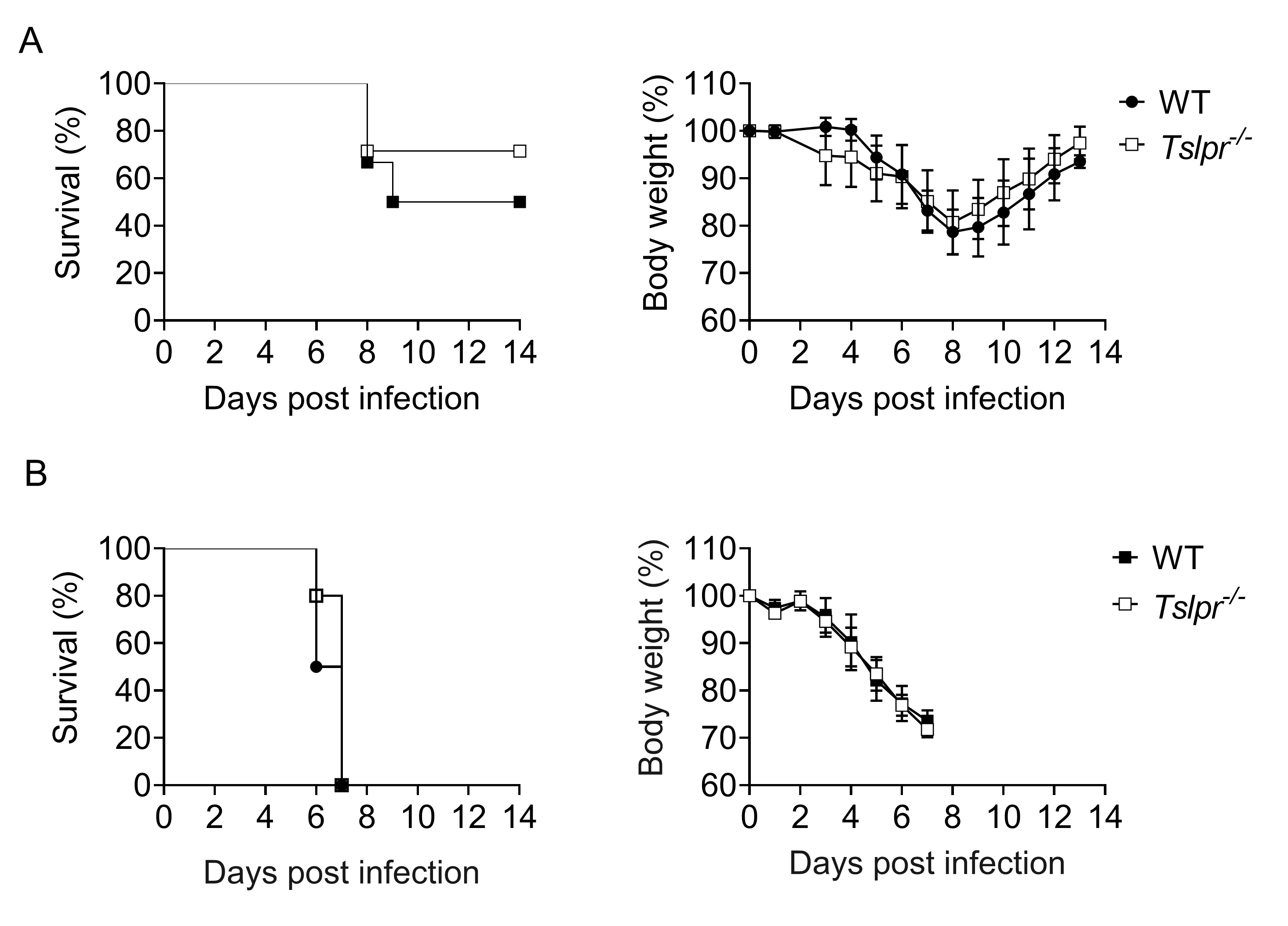

Supplement: Supplementary Figure 1 — Influenza virus susceptibility of naïve Tslpr-/- and WT mice is comparable. Naive B6-WT (n = 6) and Tslpr–/– mice (n = 6) were intranasally infected with (A) 40 PFU or (B) 100 PFU of influenza virus PR8. Weight loss and survival were monitored for 14 days. Mice were sacrificed when having lost 25% of their original body weight. Data are representative of two independent experiments and shown as mean ± SD. [file Image_1.tif]

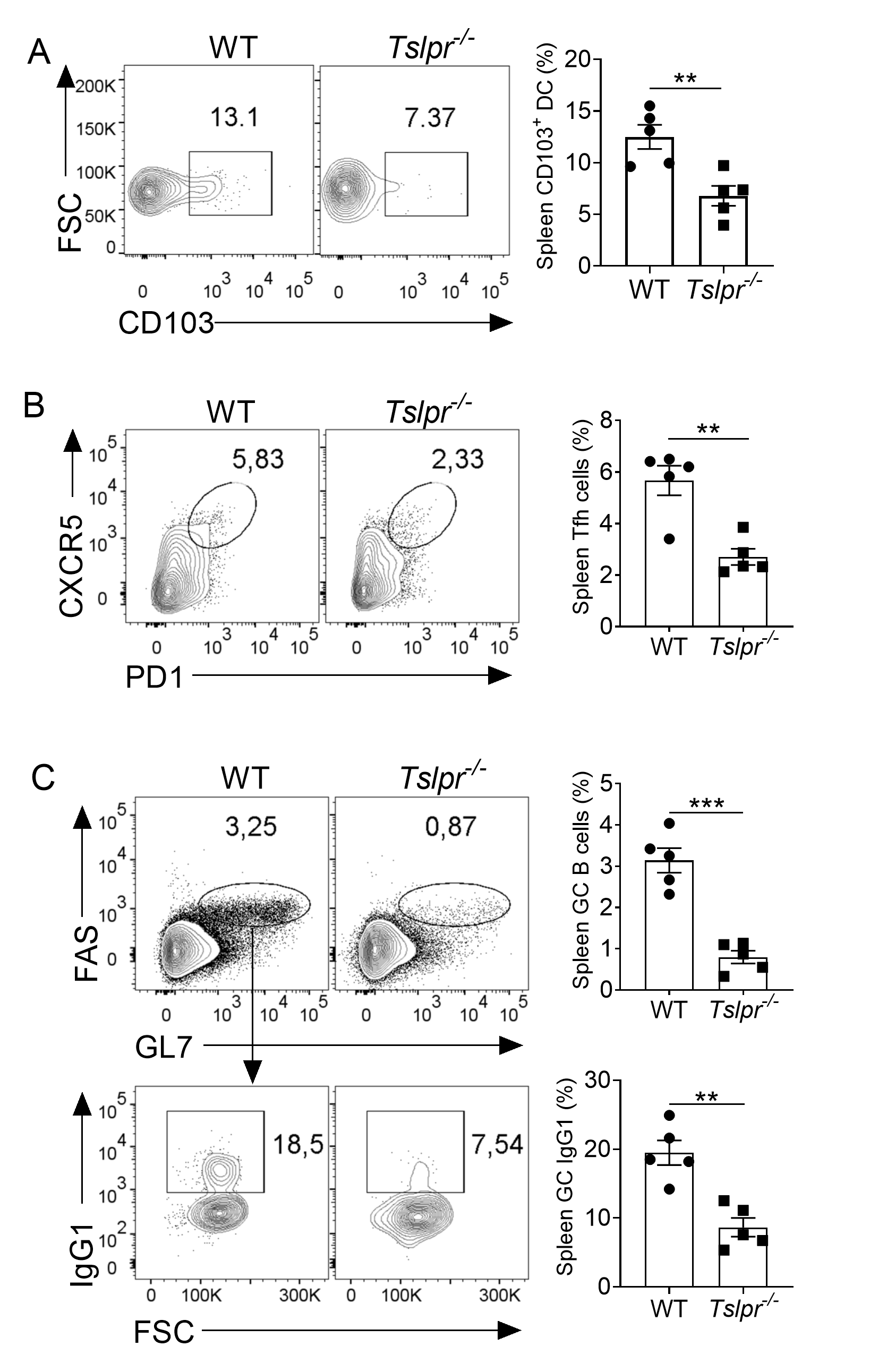

Supplement: Supplementary Figure 2 — TSLPR deficiency impairs migration of DCs and restrains GC reaction in the spleen of mice infected with live-attenuated influenza virus. (A) B6-WT (n = 5) and Tslpr–/– mice (n = 5) were intranasally infected with live-attenuated influenza virus (hvPR8-ΔNS1), and the frequency of CD103+ DCs among live CD11c+MHC-II+ immune cells in the spleen were determined at day 5 post-infection. (B, C) B6-WT (n = 5) and Tslpr–/– mice (n = 5) were intranasally infected with hvPR8-ΔNS1 and sacrificed on day 10 post-infection. The frequencies of (B) Tfh cells among live CD19−CD4+ CD44+ cells and (C, upper panels) GC B cells among live CD4-B220+ cells in spleen were determined by flow cytometry. (C, lower panels) Flow cytometry analyzing the frequency of IgG1+ GC B cells among live CD4−B220+GL7+Fas+ cells in the spleen. Data are representative of three independent experiments and shown as mean ± SEM. Each symbol represents the result from an individual animal. **P < 0.01, ***P < 0.001 by unpaired two-tailed Student’s t-test. [file Image_2.tif]

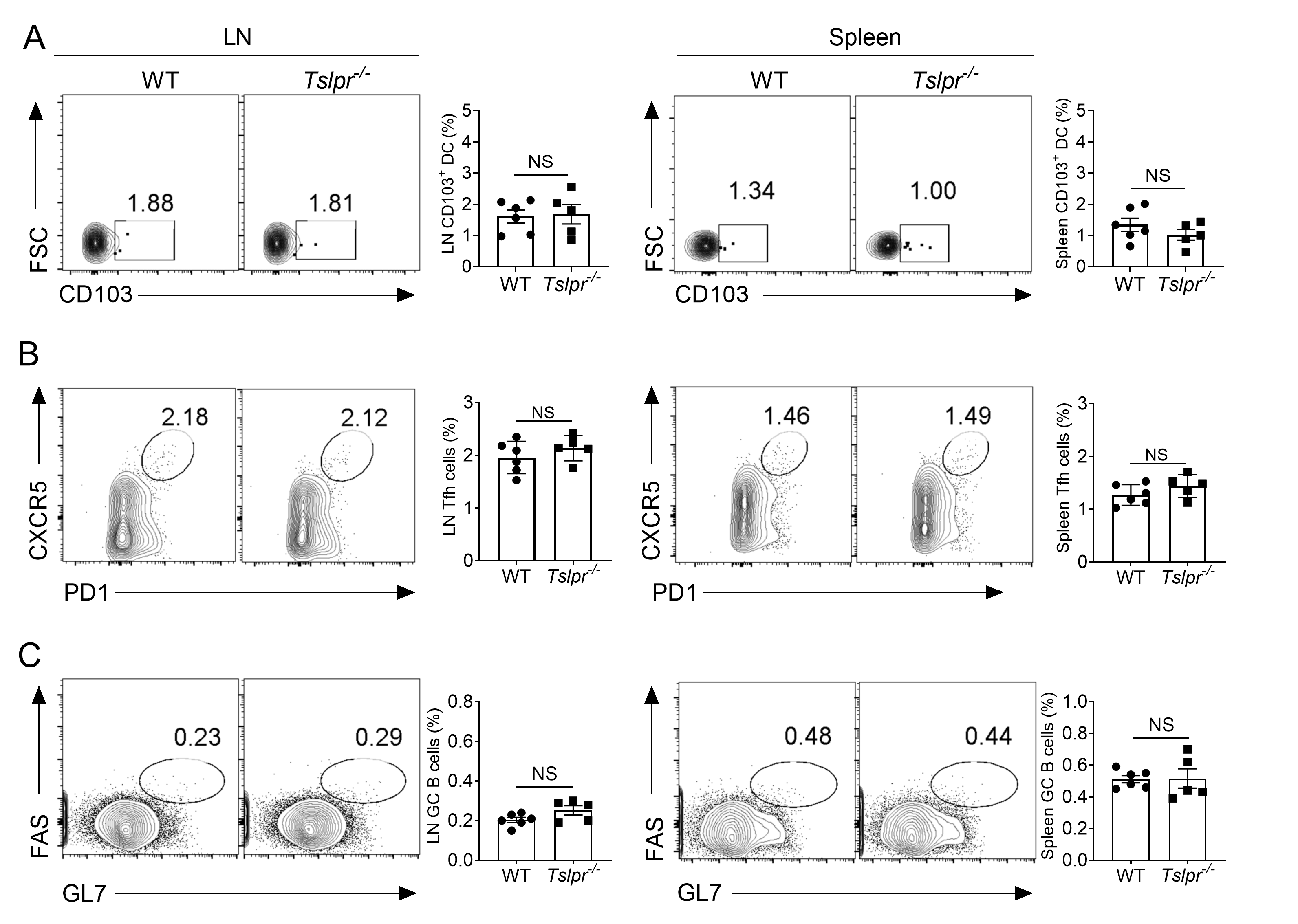

Supplement: Supplementary Figure 3 — TSLPR deficiency does not alter the proportions of migratory DCs and has no effect on the GC reaction in the lymph nodes and spleen. (A) The frequency of CD103+ DCs among live CD11c+MHC-II+ immune cells from lymph nodes and spleen was determined in naïve B6-WT (n = 6) and Tslpr–/– mice (n = 5). (B, C) B6-WT (n = 6) and Tslpr–/– mice (n = 5) were sacrificed, and the frequencies of (B) Tfh cells among live CD19−CD4+ CD44+ cells and (C) GC B cells among live CD4-B220+ cells in lymph nodes and spleen were determined by flow cytometry. Data are representative of two independent experiments and shown as mean ± SEM. Each symbol represents the result from an individual animal. Unpaired two-tailed Student’s t-tests were performed to determine significant difference. NS, no significant difference. [file Image_3.tif]
